# A nationwide dataset of stable isotopes in meteoric and terrestrial water across Peru

**DOI:** 10.1038/s41597-025-05413-x

**Published:** 2025-07-12

**Authors:** Carol Romero, James Apaéstegui

**Affiliations:** 1 Subdirección de Ciencias de la Atmósfera e Hidrósfera, Instituto Geofísico del Perú (IGP), Lima, 15012 Peru; 2 Programa de Maestría en Recursos Hídricos, Universidad Nacional Agraria La Molina (UNALM), Lima, 15024 Peru

**Keywords:** Hydrology, Atmospheric science, Palaeoclimate

## Abstract

Water Stable Isotopes (δ^18^O, δ^2^H) are valuable tools for tracing sources and interactions in the water cycle, providing important information dedicated to understanding physical mechanisms related to global climate. Despite their significance, the topic of isotopic research in South America has been hindered by limited data. To address this gap, we launched a national-level water stable isotope dataset covering different water sources in Peru (WSI-PeruDB). The dataset contains curated in-house data and incorporates previously published records from various locations collected between 2000 and 2021. The WSIPeruDB dataset is composed of 489 water collection sites and allows a comprehensive use of the dataset by implementing standardized metadata templates containing essential geographical information such as latitude, longitude, and altitude (from sea level to 5000 m a.s.l), and sampling information such as sample type (e.g. groundwater, precipitation, river, spring, and others) and sampling frequency (e.g. biweekly, daily, monthly). The WSIPeruDB dataset is publicly available on Zenodo, facilitating access and use for the scientific community.

## Background & Summary

Understanding water cycle dynamics is critical in the context of ongoing climate variability and increasing human pressures on water resources^1,2^. Stable isotopes of water (δ¹⁸O, δ²H) provide a valuable means for tracing the movement and sources of water within the hydrological cycle across a range of spatial and temporal scales. Isotopic fractionation in water elements during evaporation, condensation, and precipitation processes allows for insights related to atmospheric circulation, moisture sources^3,4^, and hydrological processes^5,6^.

In Peru, climate variability, glacier retreat, urbanization, and agricultural expansion^7^ have altered water availability and distribution, particularly in regions such as the Cordillera Blanca^8,9^ the coastal regions^10^ and Andean zones^11^. Events like El Niño–Southern Oscillation (ENSO) episodes have further influenced regional hydrological patterns^12–14^. Understanding these impacts requires detailed, spatially explicit data on water sources and flow paths. Stable isotope data, by serving as ideal geochemical tracers, support the investigation of processes not covered by conventional methods, such as precipitation sources, groundwater recharge, streamflow origin, and catchment storage. Several previous studies have contributed to the understanding of isotopic patterns in Peru and into the broader context of the Andean region^15–26^. These works underscore the relevance of stable isotope applications for understanding climate variability, water sources, and the impacts of extreme events. The dataset presented here builds on this foundation by expanding geographic coverage and including a wider array of water types and measurement periods.

Building on these applications, stable isotopes have been employed in the evaluation and refinement of general circulation models (GCMs)^27^, offering a means to compare modelled and observed isotope distributions. More recently, studies have applied stable isotopes to examine key hydrological processes associated with extreme weather events^28–30^.

Despite the global availability of water isotope data through platforms like the Global Network of Isotopes in Precipitation^31^ (GNIP) and the Waterisotopes Database^32^ (wiDB), isotopic data coverage in Peru remains sparse and inconsistently updated. This represents a significant gap for researchers and decision-makers aiming to evaluate climate-water interactions, validate hydrological and climate models, or support environmental planning.

Here, we present a comprehensive, curated dataset of water stable isotope measurements (δ¹⁸O, δ²H) from meteoric and terrestrial waters across Peru (WSIPeruDB)^33^, compiled from published studies, technical reports, theses, and global databases. This dataset aggregates observations from diverse sources, some of which are originally presented and have not been previously integrated into international repositories. By consolidating and standardizing these data, we aim to improve accessibility and enable broader reuse in hydrological, climatological, and ecological studies.

## Methods

**Fig. 1:**
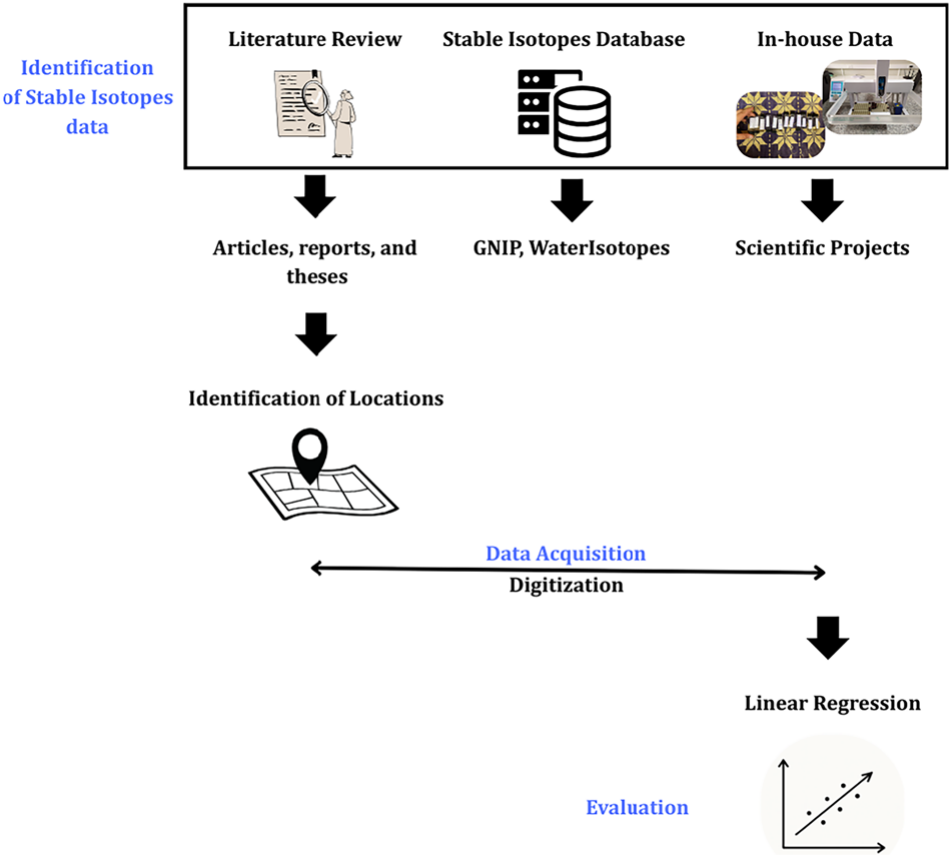
Workflow of the water stable isotopes dataset in Peru (WSIPeruDB) development.

The development of the water stable isotopes dataset followed three steps: (i) collection of the isotopic information from published data, including scientific articles, reports and theses, project partners, and public databases; (ii) the identification of isotopic station’s locations across Peru; (iii) evaluation of the stable isotopes from in-house data; (iv) creation of standardized metadata templates. The workflow is shown in Fig. 1.

### Isotopic Dataset Compilation from scientific articles, reports, theses, project partners, and public databases

We compiled the WSIPeruDB dataset through extensive retrieval and transcription of information from a variety of sources such as scientific articles, technical reports, theses, and global databases. All content was standardized using a structured metadata template (described in Table 1) to ensure consistency and usability across the entire dataset. These sources include both in-house data and external datasets, each clearly referenced in the WSIPeruDB_site_information.xlsx hosted at the WSIPeruDB Zenodo repository^33^ in accordance with their respective data-sharing policies.

**Table 1:** Template with the categories for site information.

	Description
**ID**	Station coding with water type classification based on projects. We handle the coding
**Station**	Name of the station
**Latitude**	Latitude in decimal degrees
**Longitude**	Longitude in decimal degrees
**Altitude**	Elevation above sea level in meters
**Department**	Region of the study area
**Start Date**	Start Date Time for collection of the time-integrated sample, in local time, time in 24 hr format
**End Date**	End Date Time for collection of the time-integrated sample, in local time, time in 24 hr format
**Sampling frequency**	The temporal resolution of the sample: daily, biweekly, monthly, annual
**Sample Type**	precipitation, river, spring, lake, ice, tap, mine, wetland, groundwater, soil, snow, cave drip water
**Number of δ**^**18**^**O data**	Number of δ^18^O data
**Number of δ**^**2**^**H data**	Number of δ^2^H data
**δ**^**18**^**O analytical precision**	analytical standard deviation
**δ**^**2**^**H analytical precision**	analytical standard deviation
**Contact**	Contact name
**Contact email**	Contact email
**References**	Article or page citation where the database is located
**Project_ID**	It refers to the ID used **if it is stored in another repository**
**Database**	Name or link of the database related to the project ID

Among the 489 water collection points, 17 are associated with the GNIP^31^, accessible via the International Atomic Energy Agency (IAEA) WISER platform (https://nucleus.iaea.org/wiser). While the metadata for these points is included in our dataset, users must register on the GNIP platform to access the full data, in accordance with IAEA’s terms of use. An additional 34 collection points originate from datasets hosted in the Purdue University research repository. Twelve of these are linked to the study by Welp *et al*.^34^. (https://purr.purdue.edu/publications/4121/1), and 22 to the work of Alvarez-Campos *et al*.^35^. (https://purr.purdue.edu/publications/3919/citations/1). Both datasets are openly available for academic use and do not require registration. Furthermore, we incorporated metadata from 347 isotopic collection points listed in the WaterIsotopes database^32^ managed by the University of Utah (https://wateriso.utah.edu/waterisotopes/index.html), which is also publicly accessible.

In all cases, we followed the respective data contributor policies. For all databases mentioned above, only metadata and site identifiers were included in the WSI-Peru dataset to ensure compliance with reuse restrictions. Users are directed to the original repositories for full data access when required. Each data point included in the WSIPeruDB dataset is accompanied by a clear source reference by adding the project ID and database location, as shown in Table 1, enabling full traceability.

### Identifying isotopic station locations

**Fig. 2:**
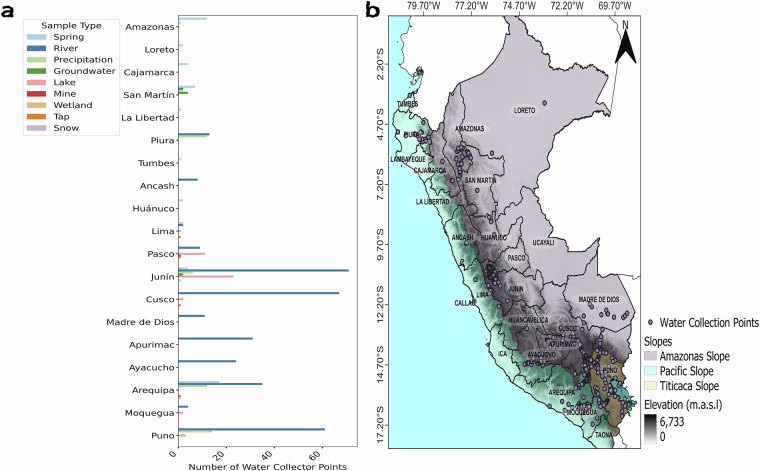
(**a**) Water collection points in Peru according to the sample type across the WSIPeruDB dataset. (**b**) Geospatial distribution of water collection points (purple circles) in Peru. Shaded areas represent the slopes: the Amazon Slope (grey color), the Pacific Slope (cyan color), and the Titicaca Slope (brown color). The topography was derived from SRTM (Shuttle Radar Topography Mission) data with a resolution of 90 meters.

To collect geographic information from the scientific article by Lambs *et al*.^26^, we used the open-source Geographic Information System (GIS) tool QGIS (Quantum GIS)^36^ to georeference the map included in the manuscript. We extracted the figure from the article and used the Layer → Georeferencer function in QGIS to georeference the image. This process generated a TIFF file, which was overlaid onto a base map of Peru. As a result, we were able to accurately determine the geographic coordinates of each isotopic station presented in the study as shown in Fig. 2b.

### Evaluation of the stable isotopes from in-house data

In-house data refer to stable isotope measurements collected by our research institution^15,16,20^ and project collaborators^17–19^. All datasets were collected following IAEA standard protocols for water sampling for isotopic analysis. Samples were stored in high-density polyethylene (HDPE) bottles to prevent evaporation. Isotopic compositions were analyzed using laser absorption spectrometers: a Picarro L2120i at the University of Brasília and a Picarro L2130i at the University of São Paulo. Post-processing was performed using the Laboratory Information Management System (LIMS) for laser-based and light-stable isotope data.

Calibration was carried out using international standards (VSMOW), and all isotopic values are reported relative to VSMOW. Analytical precision for each dataset is detailed in the supplementary file *WSIPeruDB_site_information.xlsx*, available via the Zenodo repository.

To ensure data quality, we implemented a validation procedure based on the Local Meteoric Water Line (LMWL), which characterizes the linear relationship between δ¹⁸O and δ²H in precipitation for a given location or region. Data points falling outside the 3σ range of the LMWL regression were considered outliers and excluded. This quality control procedure follows the IAEA’s recommended guidelines for isotope data processing^37^. Additionally, the resulting LMWLs were compared to the Global Meteoric Water Line (GMWL), as described by Craig^38^ (δ²H = 8 × δ¹⁸O + 10). Only data that passed this validation step were included in the WSIPeruDB dataset.

## Data Records

The dataset is publicly available through the WSIPeruDB Zenodo repository^33^ and includes stable isotope data and associated metadata from 489 water collection points across 19 regions of Peru, covering the period from 2000 to 2021. The dataset is organized into four main files; all provided in open formats to support accessibility and reuse.

1. **WSIPeruDB_site_information.xlsx.** This file contains metadata for each collection site, including station name, geographic coordinates, elevation, region, sample type, sampling period and frequency, and source project identifiers.
2. **WSIPeruDB_dataset_information.xlsx.** This file includes in-house stable isotope measurements (δ¹⁸O, δ²H, and d-excess), along with the corresponding sampling date, location data and meteorological data.
3. **WSIPeruDB_template.xlsx.** A standardized Excel template designed for future data contributions. It contains four worksheets: two for metadata descriptors (site and dataset information), and two editable sheets for users to input new collection data. Categories and field descriptions are summarized in Tables 1 and 2.

**Table 2:** Template with the categories for isotopic data information.

	Description
**ID**	Station coding with water type classification based on projects. We handle the coding
**Sample Collection Date**	Datetime of sample collection in local time
**δ**^**18**^**O**	δ^18^O data
**δ**^**2**^**H**	δ^2^H data
**Dxs**	Dxs data
**Sample Type**	precipitation, river, spring, lake, ice, tap, mine, wetland, groundwater, soil, snow, cave drip water
**Department**	Region of the study area
**Latitude**	Latitude in decimal degrees
**Longitude**	Longitude in decimal degrees
**Altitude**	Elevation above sea level in meters
**Meteorological data**	Precipitation, snowfall
4. **site_information_numeral.geojson.** A geospatial file compatible with GIS platforms, showing the spatial distribution of collection points along with relevant site metadata.

The WSIPeruDB dataset covers a wide diversity of environmental sample types. To facilitate their identification, we have assigned specific labels to the sample type: 01 for rainwater, 02 for river water, 03 for spring, 04 for lake, 05 for ice, 06 for tap, 07 for mine, 08 for wetland, 09 for groundwater, 10 for soil, 11 for snow, and 12 for cave drip water. The distribution of these sample types of our dataset can be shown in Fig. 2a. These codes are integrated into the ID using a numbering system based on project identifiers of the water collector points. This choice ensures the uniqueness of the project numbers, reducing the possibilities of changes or confusion, and provides greater scalability, especially with the expectation of new water collector point contributions in the future.

Each external dataset included in WSIPeruDB is referenced according to its original citation and detailed in the accompanying metadata file. Users interested in full datasets from external sources are directed to the original repositories, in compliance with each source’s data-sharing policy.

It is important to highlight that researchers who are interested in contributing to new water collector points are welcome to upload their data to our WSIPeruDB dataset. We encourage researchers to archive their data as it can be driven by various factors such as scientific journal requirements, grant funding obligations, or even a genuine commitment to supporting the open data initiative.

## Technical Validation

To ensure the reliability of the dataset, all in-house stable isotope measurements were subjected to a quality control procedure based on the LMWL. A linear regression was performed, and data points falling outside three standard deviations (3σ) from the regression line were identified as outliers and excluded. This method follows the quality assessment guidelines provided by the IAEA.

For external sources such as GNIP and the WaterIsotopes database, only site information as shown in Table 1 were incorporated into the WSIPeruDB dataset. No actual isotope measurement data from these sources were included. This approach is intended to facilitate users access to those databases while ensuring compliance with their respective data-sharing policies.

## Data Availability

The *WSIPeruDB* Python package is an open-source and provides tools for visualizing the linear regression of the in-house stable isotope data. The code and documentation are available on GitHub at https://github.com/karoru23/WSI-PeruDB. A public-facing visualization of the data is also accessible via the Instituto Geofísico del Perú (IGP) - institutional geospatial viewer at https://ide.igp.gob.pe/geovisor/isotopicas/. There are no restrictions on access or use.
